# Detection of Citrullinated Fibrin in Plasma Clots of Rheumatoid Arthritis Patients and Its Relation to Altered Structural Clot Properties, Disease-Related Inflammation and Prothrombotic Tendency

**DOI:** 10.3389/fimmu.2020.577523

**Published:** 2020-12-04

**Authors:** Johannes A. Bezuidenhout, Chantelle Venter, Timothy J. Roberts, Gareth Tarr, Douglas B. Kell, Etheresia Pretorius

**Affiliations:** ^1^ Department of Physiological Sciences, Faculty of Science, Stellenbosch University, Stellenbosch, South Africa; ^2^ Department of Biochemistry and Systems Biology, Institute of Systems, Molecular and Integrative Biology, Faculty of Health and Life Sciences, University of Liverpool, Liverpool, United Kingdom; ^3^ Department of Clinical Epidemiology, University College London Hospital NHS Foundation Trust, London, United Kingdom; ^4^ Division of Rheumatology, Department of Medicine, Faculty of Medicine and Health Sciences, Stellenbosch University, Cape Town, South Africa; ^5^ The Novo Nordisk Foundation Centre for Biosustainability, Kemitorvet, Technical University of Denmark, Kongens Lyngby, Denmark

**Keywords:** rheumatoid arthritis, fibrinogen, coagulation, citrullination, cardiovascular risk

## Abstract

**Aims:**

The risk of cardiovascular events in patients with Rheumatoid Arthritis (RA) is disproportionately heightened as a result of systemic inflammation. The relative effect of autoimmune-associated citrullination on the structure and thrombotic potential of fibrin(ogen) remains unknown. We therefore compared indices of vascular function, inflammation, coagulation and fibrin clot composition in RA patients with healthy controls and evaluated parameter association with disease presence.

**Methods:**

Blood samples were collected from 30 RA patients and 30 age- and gender-matched healthy volunteers. Levels of serum amyloid A (SAA), c-reactive protein (CRP), soluble intercellular adhesion molecule 1 (sICAM-1) and soluble vascular cell adhesion molecule 1 (sVCAM-1) was measured using a sandwich immunoassay. Whole blood coagulation was assessed using Thromboelastography (TEG^®^). Fibrin clot networks and fiber structure was investigated using Scanning Electron Microscopy. The detection and quantification of citrullination in formed fibrin clots was performed using a fluorescently labeled Citrulline monoclonal antibody with Fluorescence Wide Field Microscopy.

**Results:**

Concentrations of SAA, CRP and ICAM-1 were significantly elevated in RA patients compared to controls. TEG parameters relating to coagulation initiation, rate of fibrin cross-linking, and time to reach maximum thrombus generation were attenuated in RA patients. Microscopic analysis revealed denser networks of thicker fibrin fibers in RA patients compared to controls and multiple citrullinated regions within fibrin clot structures in RA patients were present.

**Conclusion:**

Our findings provide novel evidence for the citrullination of fibrin within vasculature is more prominent in RA plasma compared to control plasma and plasma is more accessible than synovial fluid. Citrullinated fibrinogen could play a role as a determinant of thrombotic risk in RA patients.

## Introduction

Rheumatoid Arthritis (RA) is a chronic, systemic autoimmune disease characterized by both peripheral joint and extra-articular site inflammation, with an increased predisposition to a higher incidence of cardiovascular disease (CVD) (1). CVD is almost 50% more common in RA patients than the general population and is the most frequent cause of early mortality (2). Traditional risk factors for CVD (age, hypertension, obesity, etc.), do not fully account for the elevated occurrence of CVD events, and thus RA (genetics and disease characteristics) has been identified as a strong independent risk factor (3). The interdependence of inflammatory and hemostatic pathways is well established and observable in multiple types of tissue, organs and pathologies (4). Disruption of the tightly regulated homeostatic control of immune and hemostatic systems could result in a rapid progression towards a prothrombotic tendency, a central cause of ischemic stroke and myocardial infarction (5). This circumstance holds true for RA, with elevated levels of both pro-inflammatory and prothrombotic markers (e.g. D-dimer Fibrinogen Tissue Factor (TF) and von Willebrand factor (vWF)), which is associated with one another and with the risk of future cardiovascular complications (6).

Key intermediaries of this manifestation are the structural components of formed thrombi. Soluble fibrinogen is cleaved by thrombin in order to form dense matrices of thin fibrous protein known as fibrin (7). Polymerized fibrin networks are essential for wound healing and other occlusive physiological processes (7). However, exposure to inflammatory biomarker stimuli (such as CRP (8), SAA (9), and pro-inflammatory cytokines) can result in the alteration of mechanical and viscoelastic properties of fibrin clots into a prothrombotic phenotype. This phenomenon has previously been observed in RA plasma clots (10, 11). Various immunopathogenic processes related to RA development can exert upstream amplification of the coagulation cascade as well as impairing fibrin clot dissolution (12).

**Figure 1:**
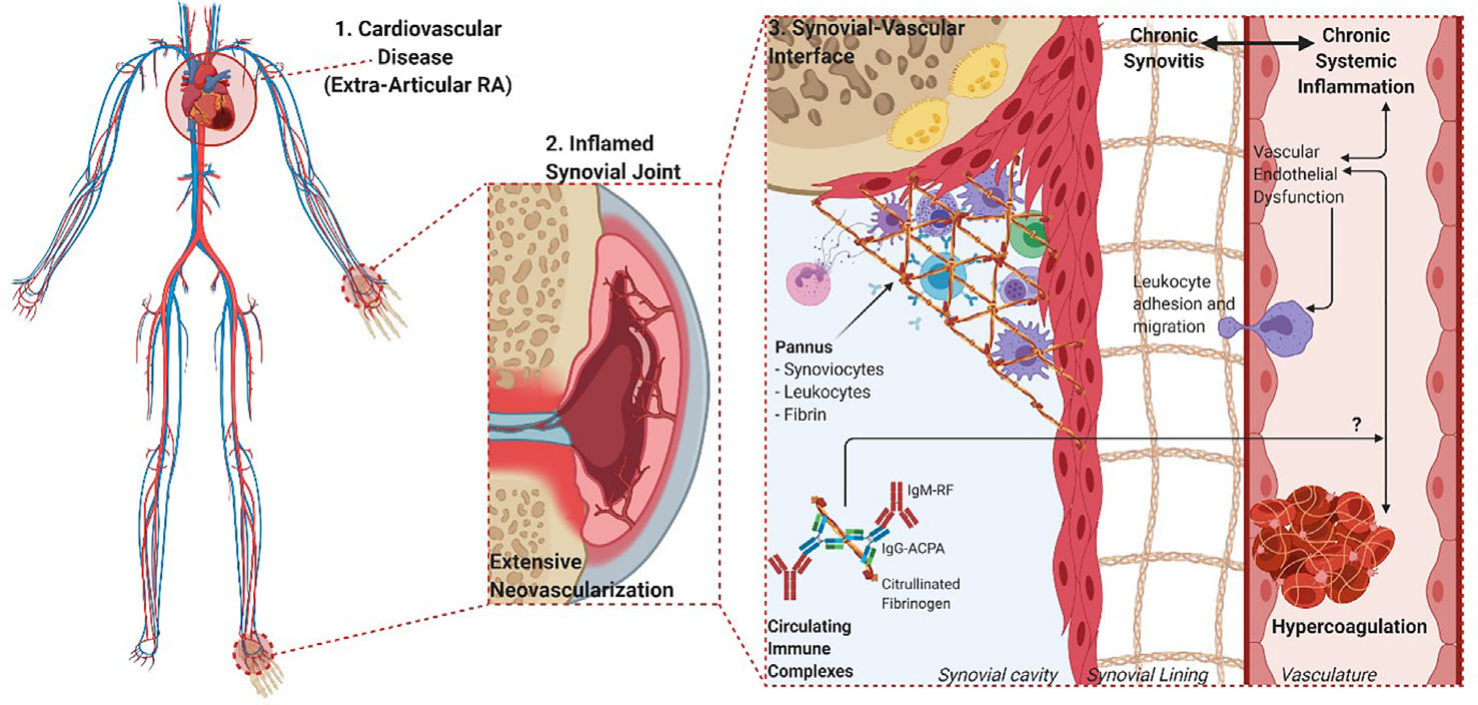
Overview of the overlapping processes of inflammation and coagulation in both synovial and vascular compartments. 1. The chronic and systemic nature of the inflammatory response in RA characterizes the disease as an independent risk factor for CVD. 2. The movement of leukocytes, inflammatory cytokines, procoagulant factors and immune complexes are aided by vascular endothelial dysfunction and neovascularization of hyperproliferative joint tissues. 3. The role of fibrin(ogen) is integral to the formation of hyperplastic and destructive synovial tissue (pannus) and vascular thrombosis, while being a prominent self-protein target of aberrant citrullination and autoimmunogenicity in RA (Created with BioRender.com).

Fibrin(ogen) is also a potent pro-inflammatory signaling entity itself, mainly through ligand-receptor interactions with immune cells that further propagates pro-inflammatory effects (13). The deimination of arginine residues in fibrin(ogen) to yield non-charged citrulline residues is a distinctive RA posttranslational modification. Citrullination alters normal protein structure and function that confers antigenicity to modified proteins in RA (14). The functional relationship between citrullination and the presence of a prothrombotic fibrin clot phenotype is still poorly understood. Some studies have shown that citrullination of fibrinogen prevents thrombin digestion and subsequent fibrinogenesis (15, 16). However, the experimental conditions upon which these findings are based do not reflect physiological coagulation and is inconsistent with a predominantly hypercoagulable state seen in RA (17). Inflammation-induced fibrin formation is equally present in RA synovial spaces as it is in circulation (refer to overview of processes in **Figure 1**).

Endothelial tissue dysfunction is a key process that facilitates this ubiquitous distribution of aberrant fibrin deposition in both synovia and vasculature. This pathophysiological state is characterized by the expression of cell adhesion molecules [intercellular cell adhesion molecule-1 (ICAM-1) and vascular cell adhesion molecule-1 (VCAM-1)], pro-inflammatory cytokines and pro-thrombotic markers (18, 19). This allows for the recruitment, translocation and propagation of inflammatory and thrombotic mediators across the synovial barrier (20).

There is significant overlap in inflammatory pathways responsible for joint damage in RA and hypercoagulation, coupled with the fact that disease severity has been correlated to more adverse cardiovascular complications (21). It is therefore important that these processes be examined systemically in RA, and not isolated to either vascular or synovial compartment. The aim of this study was to examine the extent to which the coagulation profiles and fibrin network architecture of RA patients are influenced by acute phase inflammation, endothelial dysfunction an autoimmune-related protein modification.

## Materials and Methods

### Ethical Considerations

Ethical approval for this study was given by the Health Research Ethics Committee (HREC) of Stellenbosch University (reference number: 6983). This study was carried out in strict adherence to the International Declaration of Helsinki, South African Guidelines for Good Clinical Practice and the South African Medical Research Council (SAMRC) Ethical Guidelines for research. Written consent was obtained from all participants (RA patients and healthy participants) prior to any sample collection.

### Study Population

The RA sample group consisted of 30 patients (24 female and 6 male) that visited the Winelands Rheumatology Clinic (Stellenbosch, South Africa) for routine check-ups. All patients fulfilled the 2010 American College of Rheumatism/European League against Rheumatism (ACR/EULAR) classification criteria for RA diagnosis (22). The median age of RA group was 53.5 years (range 22–75 years) with a mean disease duration of 10.5 years (range 1–39 years). RA participants were excluded from the study if they presented with other severe comorbidities (such as cancer or diabetes), existing cardiovascular disease or taking anticoagulant medication. RA participants were not excluded on the basis of any antirheumatic drug treatment or the use of glucocorticosteroids. The majority of RA patients (87%) were on a schedule of non-biologic disease modifying antirheumatic drugs (DMARDS, such as methotrexate, hydroxychloroquine, sulfasalazine, or leflunomide), while a lower proportion of patients were on biologic DMARDs (60%) and cortisone (14%, 5–10 mg dosage). The control group consisted of 30 age- (median 50 years, range 28–79 years) and gender- (22 female and 8 male) matched volunteer blood donors. The inclusion criteria for healthy controls were: (i) no history of thrombotic disease or inflammatory conditions (ii) no use of any chronic medication (ii) no use of anticoagulant therapy (iii) non-smokers (iv) females not taking contraceptive medication or hormone replacement therapy (v) females that are not pregnant or lactating. All demographic information is summarized in **Table 1**.

**Table 1:** Demographic and clinical characteristics of study participants.

Risk Factor	RA (n=30)	Control (n=30)
Age (years)	53.5 (22–75)	50 (28–79)
Women [n (%)]	24 (80%)	22 (73.3%)
Smokers [n (%)]	3 (10%)	–
Disease duration (years)	6.5 (1–39)	–
Hypertension [n (%)]	5 (16.7%)	–
Hypercholesterolemia [n (%)]	3 (10%)	–
Disease activity (DAS-28) classification [n (%)] - Remission - Low - Moderate - High	1 (3.3%) 17 (56.7%) 4 (13.3%) 8 (26.7%)	–
Autoantibody seropositivity [n (%)] - RF^-^/Anti-CCP^-^ - RF^+^/Anti-CCP^-^ - RF^-^/Anti-CCP^+^ - RF^+^/Anti-CCP^+^	1 (3.3%) 6 (20%) - 23 (76.7%)	
Conventional DMARD use [n (%)]	26 (86.7%)	–
Biologic DMARD use [n (%)]	18 (60%)	–
Cortisone use [n (%)]	13 (43%)	–

Values expressed as median and interquartile range (IQR) unless otherwise stated. Disease activity scores (DAS-28), were categorized in RA patients as remission, low, moderate or high (23). For full demographic information and specified list of disease modifying anti-rheumatic drugs (DMARDs) please refer to full study database in supplementary material. RF, rheumatoid factor; CCP, cyclic citrullinated peptide.

### Blood Sampling

Whole blood (WB) samples were collected in BD Vacutainer^®^blood collection tubes using 3.8% sodium citrate as anticoagulant (369714, Becton, Dickinson and Company Franklin Lakes, NJ, USA). Blood drawing on all participants was performed by a qualified nurse, or phlebotomist by sterile puncture of the antecubital vein. Blood tubes were incubated at room temperature for a minimum duration of 30 min prior to the commencement of any whole blood analysis. In order to obtain platelet poor plasma (PPP), sodium citrated blood tubes were centrifuged at 3000 × *g* for 15 min, aliquoted and stored at −80°C until further analysis.

### Thromboelastography^®^

Analysis of dynamic coagulation kinetics were performed on RA and control WB by means of Thromboelastograph^®^ (TEG^®^) 5000 Haemostasis Analyzer System (07-033, Haemonetics^®^, Niles, IL, USA). In brief, coagulation is initiated by recalcification of 340 µl WB with 20 µl of 0.2 mM Calcium chloride (CaCl_2_) (Haemonetics^®^, 7003) in a disposable TEG^®^ cup (Haemonetics^®^, 6211). Various kinetic clotting parameters are determined by assessing the resistance that the forming thrombus provides against the oscillating pin of the instrument (measuring at 37°C). Parameters derived from the thromboelastograph tracing consist of: reaction time (R, time from test start to initial fibrin formation in minutes), kinetics [K, time required to reach an amplitude (clot thickness) of 20 mm, in minutes], alpha angle (α, rate of fibrin accumulation indicated by degrees), maximal amplitude (MA, maximum strength of formed clot in millimeters) (24). Additionally, parameters of clot formation determined by the changes in the elastic modulus (G, in dynes.cm^−2^) were also included: maximum rate of thrombus generation (MRTG, in dynes.cm^−2^.s^−1^), time to maximum rate of thrombus generation (TMRTG, in minutes) and total thrombus generation (TTG, in dynes.cm^−2^) (25).

### Scanning Electron Microscopy

The ultrastructure of fibrin networks and individual fibrin fibers were examined using scanning electron microscopy (SEM). In summary, clots were prepared from thawed PPP samples of RA patients (n=10) and controls (n=10) by addition of 5 µl human thrombin (provided by South African National Blood Service, final concentration 7 IU/ml) to 10 µl PPP on a glass coverslip and transferred to a 24-well plate. Preparation consisted of washing with 1× Gibco^®^ phosphate-buffered saline (PBS, pH 7.4) (10010015, ThermoFisher Scientific, Waltham, MA, USA), chemical fixation with 4% formaldehyde (FA) (P6418, Sigma-Aldrich, St. Louis, MO, USA) and then 1% Osmium Tetrahydroxide (OsO_4_) (Sigma-Aldrich, 75632), followed by dehydration with increasing grades of ethanol and 99.9% Hexamethyldisilizane (HMDS) (Sigma-Aldrich, 37921) [for detailed protocols please refer to (24)]. Samples were carbon coated using a Quorom Q150T E carbon coater (Quorom Technologies, Lewes, UK). Plasma fibrinogen concentrations of samples used to conduct SEM analysis were determined by an independent pathology laboratory. Images were captured at an electron high tension (EHT) of 1 kV using a high resolution InLens detector of the Zeiss Merlin™ (Gemini II) FE SEM (Carl Zeiss Microscopy, Munich, Germany). Fibrin fiber diameters representative of each respective sample group (RA and control) was determined by means of image analysis software ImageJ (Version 1.52p). Three representative micrographs (78,98 µm^2^ image size, 10,000× magnification) were calibrated to scale and overlaid with a non-destructive grid (2 µm^2^ tile size). Single representative fibrin fibers were measured in 28 tiles per image, producing 84 fiber diameter measurements per sample.

### Vascular Injury Panel Analysis

Plasma concentrations of soluble ICAM-1, VCAM-1, CRP, and SAA were measured by sandwich immunoassay (K15198D, Meso Scale Diagnostics, Rockville, MD, USA). RA (n=30) and control (n=30) PPP samples and reagents were prepared as per manufacturer’s protocol. Samples were run in duplicate and measurements read on an MSD Discovery Workbench 4. Analyte concentrations were calculated from the calibration curve generated by absorbance measurements of manufacturer supplied calibrator standards.

### Immunofluorescence Microscopy

In order to determine the extent of protein deamination in fibrin networks, PPP aliquots of RA samples (n=10) and control samples (n=10) were thawed and fibrin clots prepared (refer to SEM method) on glass microscope slides in a dark room. Samples were fixed with 4% FA, washed 3× with PBS, and blocked with 5% Goat serum solution (ab7481, Abcam, Cambridge, UK) for 30 min. Clots were then stained with a 1:50 dilution Citrulline Monoclonal Antibody (2D3.1) (MA5-27573, ThermoFisher Scientific) and incubated for 1 h. Following another 3× PBS wash to remove unbound antibodies, samples were then stained with 1:500 dilution Goat Anti-Mouse IgG Secondary antibody conjugated to AlexaFluor 488 (A327273, ThermoFisher Scientific) and incubated for 1 h. Slides were washed 3× with PBS to remove unbound antibody, allowed to dry, and mounted with a glass coverslip. Samples were viewed with a Zeiss Axio Observer 7 Microscope with a Plan-Apochromat 63x/1.4 oil DIC M27 objective (Carl Zeiss Microscopy, Munich, Germany). The excitation wavelength for AlexaFluor488 was set at 450 to 480 nm and the emission wavelength at 499 to 529 nm. Three representative micrographs per sample were analyzed for relative mean fluorescent intensity (MFI) using ImageJ (Version 1.52p). Images were calibrated to scale, and a global threshold (20 pixel cut-off) applied to all analyzed micrographs.

### Statistical Analysis

**Figure 2:**
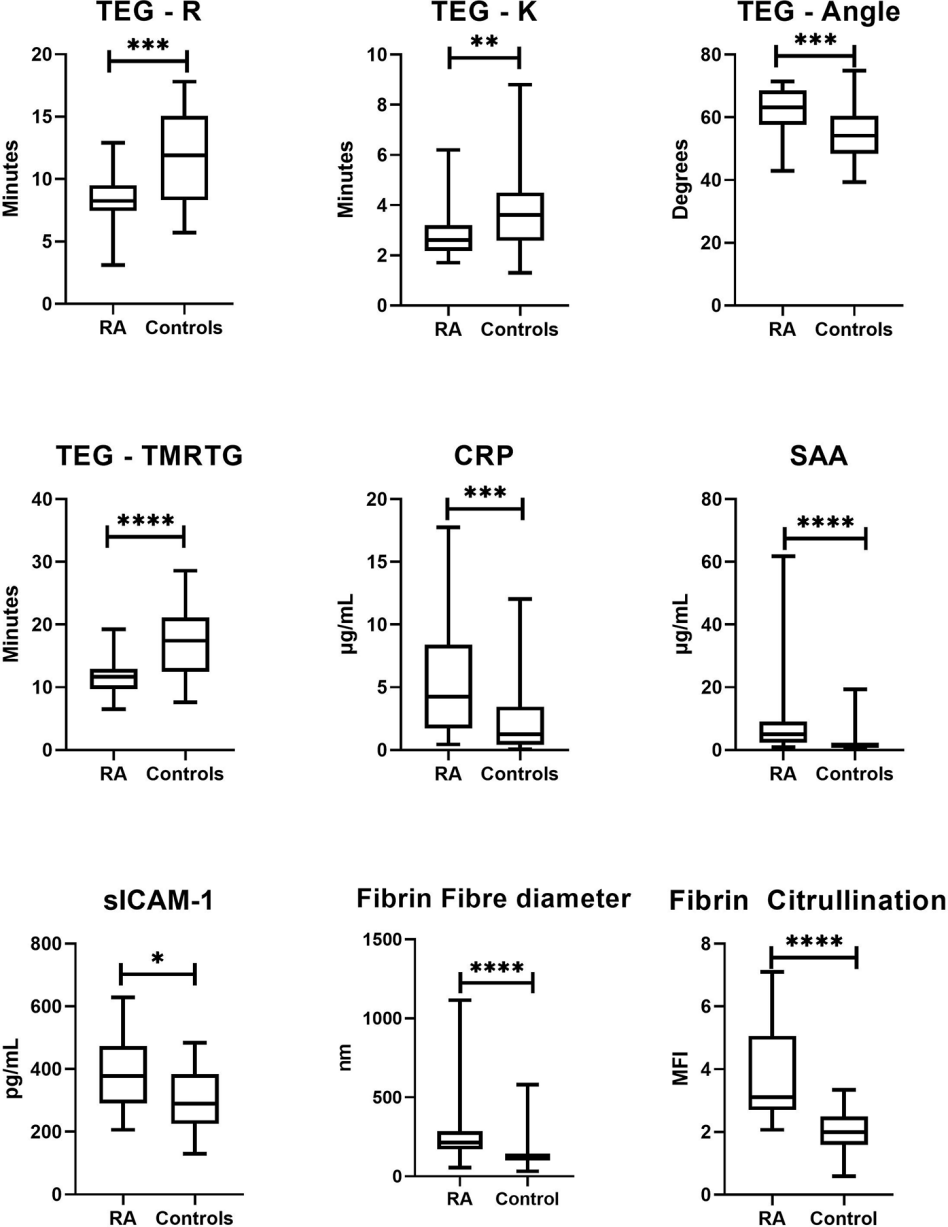
Box-and-whisker plots comparing sample distributions for each significantly altered study variable compared between RA patients and healthy controls. The level of statistical significance is indicated by the bars above each plot (*p < 0.05, **p < 0.01, ***p < 0.001, ****p < 0.0001). Viscoelastic coagulation analysis by TEG^®^ indicated that RA patients had shortened phases of clot formation initiation (R: p = 0.0005; K: p = 0.01), overall clot formation (TMRTG: p < 0.0001) and rate of fibrin deposition (α-Angle: p = 0.0003). Microscopic analysis of fibrin networks revealed that fibrin fiber diameter (p < 0.0001) and fibrin citrullination (p < 0.0001) were drastically elevated in plasma clots RA patients. Plasma levels of CRP (p = 0.001), SAA (p < 0.0001) and sICAM-1 (p = 0.01) were also significantly increased in RA patients.

Statistical analysis was performed using R version 4.0. Specifically, univariate logistic regression was performed to determine odds ratios (OR) for experimental variables using the logistic model in the rstanarm package (with default priors). ORs and 95% confidence intervals were extracted in the corresponding unit system (i.e. not z-scaled) for all variables except Fibrin fiber diameter and citrulline MFI shown in **Figure 2** which *are* z-scaled to aid interpretation. **Table 2** shows the ORs after adjustment for age and gender with unadjusted analysis identifying the same significance and effect sizes. In addition, results for classical statistical tests are reported as follows. The distribution of sample datasets for each variable experimental was determined using the Shapiro-Wilk test. Accordingly, *p*-values for each variable comparing RA to healthy controls were calculated using either a Mann-Whitney *U* test for nonparametric data or a Student t-test for parametric data. Statistical significance was set at *p*<0.05. One can see close alignment between all these and the OR results.

**Table 2:** Descriptive statistics and logistic regression for study parameters.

Parameter	RA	Control	Adjusted logistic regression
Vascular injury panel (RA n=30, Control n=25)			
CRP (μg/ml)	4.25 [1.88–8.11]	1.26 [0.43–3.15]	1.29 (1.09–1.62)*
SAA (μg/ml)	4.98 [2.56–8.84]	1.52 [0.635–2.24]	1.25 (1.08–1.55)*
sICAM-1 (ng/ml)	378 [296–460]	290 [225–366]	1.01 (1.00–1.01)*
sVCAM-1 (ng/ml)	360 [283–394]	326 [254–423]	1.00 (0.995–1.007)
Thromboelastography (RA n=30, Control n=30)			
R (min)	8.25 [7.63–9.40]	11.9 [8.50–14.7]	0.675 (0.528–0.827)*
K (min)	2.60 [2.20–3.18]	3.60 [2.60–4.38]	0.539 (0.308–0.847)*
α (°)	63.2 [58.0–68.2]	54.2 [48.5–59.8]	1.15 (1.07–1.27)*
MA (mm)	55.9 [52.7–61.4]	58.9 [54.7–63.9]	0.960 (0.889–1.03)
MRTG (dyn·cm^−2^·s^−1^)	5.01 [3.90–5.96]	4.23 [3.28–5.70]	1.03 (0.767–1.40)
TMRTG (min)	11.7 [9.79–12.8]	17.4 [12.6–21.0]	0.737 (0.607–0.858)*
TTG (dyn·cm^−2^)	620 [519–756]	719 [607–884]	0.997 (0.995–1.00)
Microscopy analysis (RA n=10, Control n=10)			
Fibrin fiber diameter (nm)	214 [170–285]	120 [100–144]	22.7 (17.1–31.4)*
Citrullinated fibrinogen (MFI)	3.11 [2.72–5.01]	1.99 [1.64–2.45]	46.1 (7.81–419)*

Parameter values for RA and control are expressed as median [IQR]. Odds ratios for age- and gender-adjusted logistic regression model is listed with the 95% confidence interval. *Denotes ORs of variables that have statistically significant predictive value within the logistic regression model.

## Results

### Subjects

Demographic information of all study participants is listed in **Table 1**. The RA sample group closely resembles the general population distribution for age (median: 54 years) and sex (80% female) of the disease (26). The control group of healthy volunteers was closely matched to the RA group with regards to age (median: 50 years) and sex (73% female). The RA sample group was heterogeneous with respect to clinical presentation, with most patients on an anti-rheumatic drug therapy regime. The majority of RA patients also presented with positive titers for anti-cyclic citrullinated peptide (CCP) (77%) and rheumatoid factor (97%) autoantibodies.

### Confirmation of Altered Inflammatory and Vascular Function Profile in RA

Circulating concentrations of endothelial function and acute phase markers are shown in **Table 2**, with significantly altered markers graphically presented in **Figure 2**. As expected, all markers were elevated in RA compared to controls [CRP (median 4.25 µg/ml vs 1.26, p=0.001), SAA (4.98 µg/ml vs 1.52, p<0.0001), sICAM-1 (378 ng/ml vs 290, p=0.01), sVCAM-1 (360 ng/ml vs 326, p=0.9)]. Univariate logistic regression also indicated that levels of CRP, SAA and ICAM-1 were significantly predictive of disease status (the nominal variable defined as the presence of absence of RA, represented by healthy controls), as shown by both the ORs listed in **Table 2**.

### Functional Coagulation Assessment Indicates a Prothrombotic Tendency in RA

Whole blood coagulation parameters as measured by TEG^®^ are listed in **Table 2**, with significantly altered parameters illustrated as box-and-whisker plots in **Figure 2**. RA patients showed significantly altered rates of clot formation compared to healthy controls. This included shortened clot initiation (R; OR=0.675, p=0.0005 and K; OR=0.539, p=0.01), augmented fibrin cross-linking (α; OR=1.15, p=0.0003) and shortened time to maximal thrombus formation (TMRTG; OR=0.737, p<0.0001). Measures of overall clot strength (MA) and growth (TTG) were attenuated in RA but did not statistically differ from those of controls.

### Scanning Electron Microscopy Analysis Exposes Anomalous Fibrin Network Architecture in Formed RA Clots

**Figure 3:**
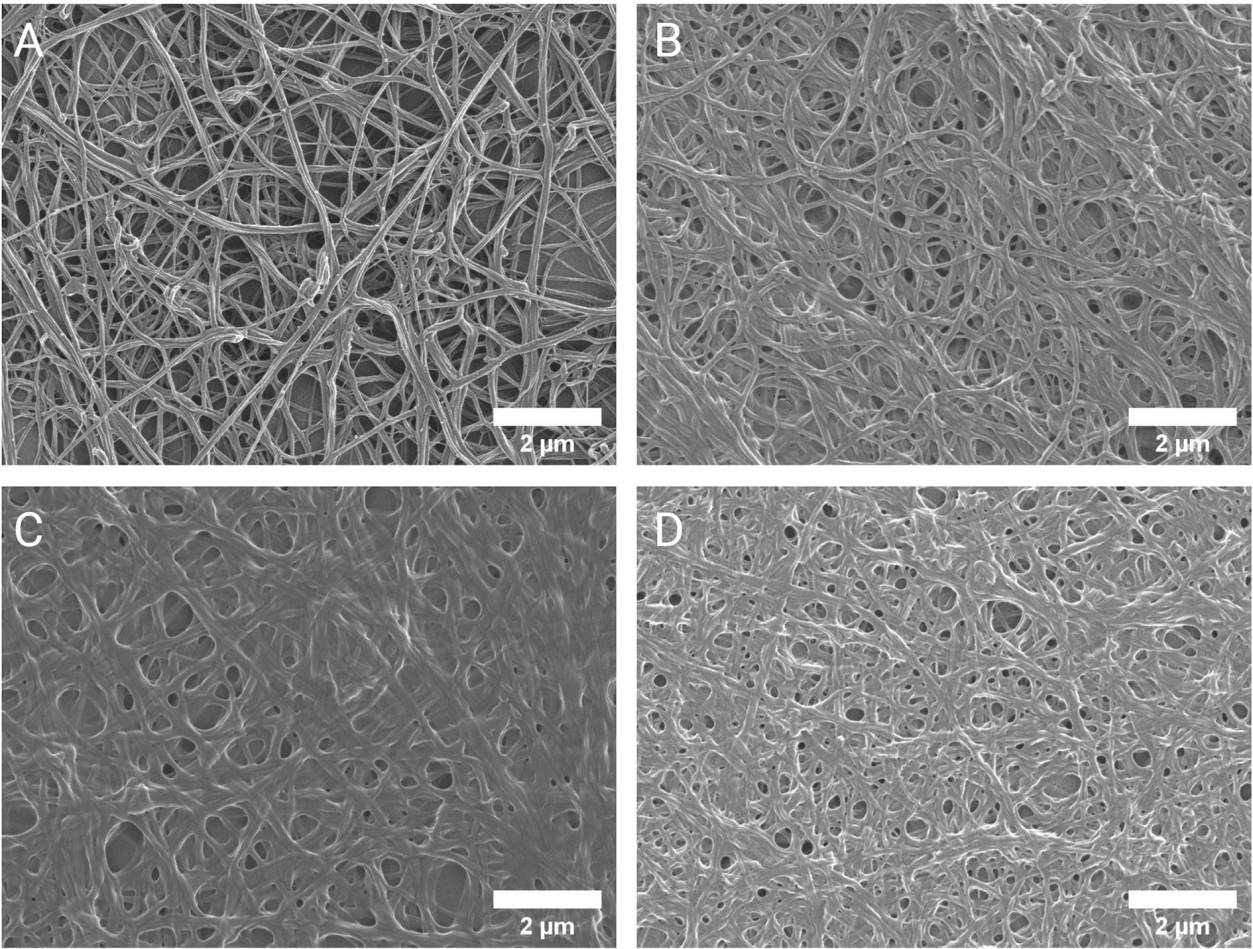
Scanning electron micrographs of the fibrin network ultrastructure. Representative micrographs of the fibrin network in healthy controls **(A)** and RA patients **(B–D)**. The altered clot ultrastructure in RA, consisting of less permeable networks of thicker fibrin fibers, represents a prothrombotic phenotype.

Further investigation into the apparent modification of the clot structure in RA was carried out by means of SEM. Results (refer to **Figure 2**) indicate that fibrin fiber diameters in representative areas were significantly increased in RA versus controls (median diameter 214 nm vs 120 nm, p<0.0001). Examining the networks qualitatively, it is evident that *ex vivo* formed clots from RA samples have denser, less porous fibrin networks. **Figures 3B** to **D**, representative of the RA sample group, illustrates the amalgamation of fibrin monomers that contribute to increased fibrin fiber diameter and overall network density. This contrasts sharply with the ultrastructural attributes of **Figure 3A** (Healthy control sample), which demonstrates thinner protein strands and a more permeable fibrin network. Mean levels of fibrinogen did not significantly differ between RA patients and healthy controls used for SEM imaging (median 3.15 vs 2.95 g/L, p=0.547). This may suggest that differences observed in the fibrin network structure and thromboelastic profiles of RA patients could be attributed to factors independent from direct influences of clot formation such as fibrinogen and thrombin concentration.

### Fluorescence Microscopy Analysis of Plasma Clots Reveals a Higher Number of Citrullinated Sites in RA Fibrin Networks Compared to Controls

**Figure 4:**
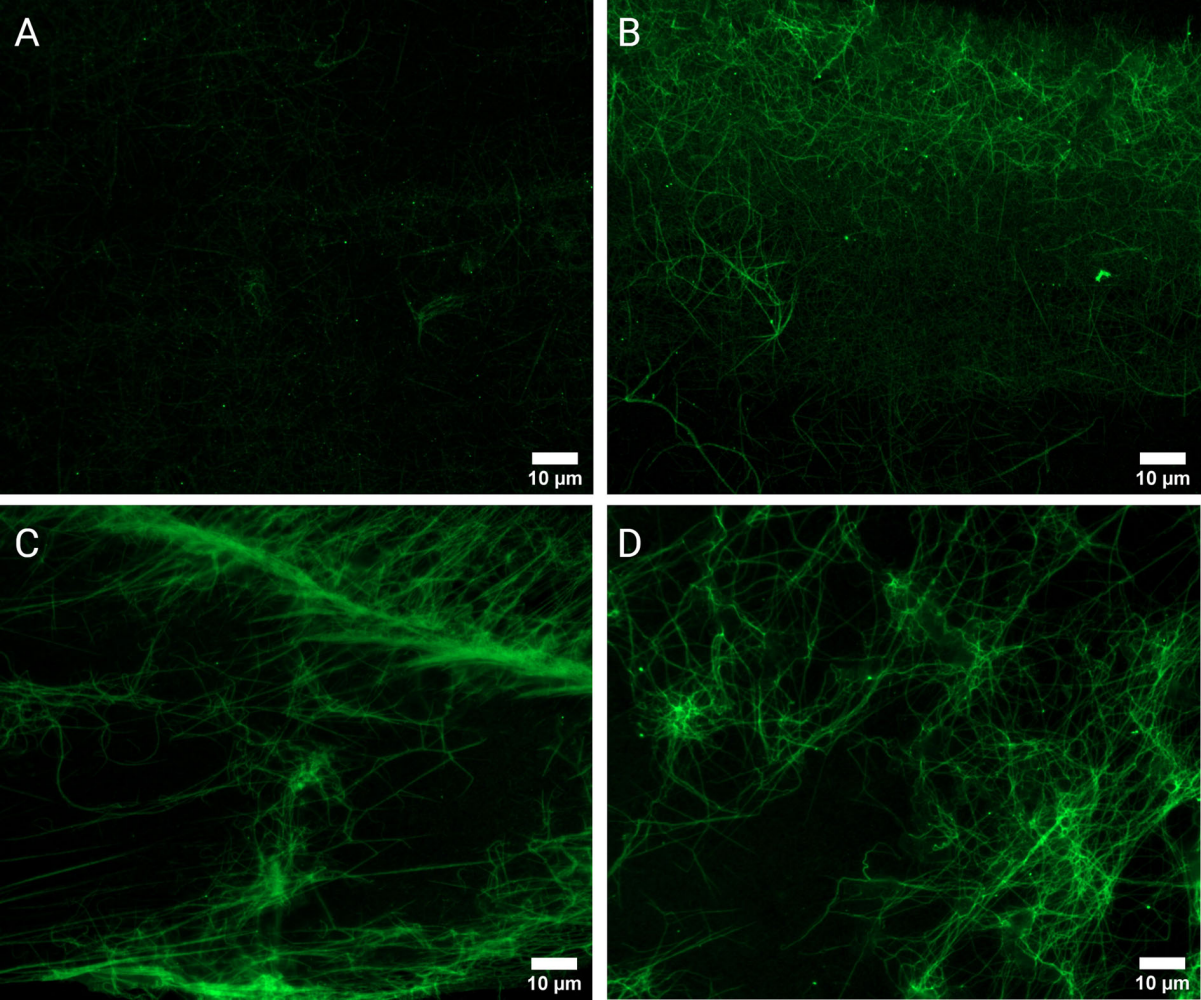
Fluorescence microscopy of PPP clots stained with a Citrulline monoclonal antibody (green). Micrographs of representative control **(A)** and RA samples **(B–D)**. Analysis confirmed the observable presence of enhanced fluorescent signal in RA samples (n=10) versus healthy controls (n=10).

To investigate the presence and extent of potential citrullination in RA (n=10) and Control (n=10) PPP thrombi, fluorescence analysis using a Citrulline-identifying monoclonal antibody with immunofluorescence microscopy was performed (**Figure 4**). Acquired image data (**Figure 2**) suggests a higher degree of citrullinated fibrin fluorescence in RA fibrin networks versus controls (median MFI 3.88 vs 2.02, p<0.0001), with logistic regression analysis (**Table 2**) showing that fibrinogen citrullination is associated with RA to a substantial degree (Adjusted OR=46.1, 95% CI: 7.81–419).

## Discussion

There is a need to bridge translational gaps between RA immunopathogenesis and systemic vascular and hemostatic irregularities. The link between RA autoimmune patterns and its possible role in exacerbating thrombosis is still poorly understood.

Crosstalk between immune and hemostatic systems with the endothelium represents a critical interface in which both arthritic and cardiovascular pathologies are initiated and propagated. We therefore analyzed a panel of biomarkers that are representative of this dynamic milieu and is associated with RA disease severity and CVD. Levels of both acute phase reactants (CRP and SAA) were significantly elevated in RA patients (**Figure 2**) and showed a strong association with the disease (**Table 2**). This was expected as acute phase reactant concentrations rise dramatically under acute inflammatory states, with both CRP and SAA shown to reliably predict disease severity and CVD risk in RA (27). Both molecules also have demonstratable prothrombotic cellular effects by influencing endothelial and peripheral blood mononuclear cell expression of coagulation factors. Increased levels of soluble cell adhesion molecules (sICAM-1 and sVCAM-1) indicate endothelial dysfunction that facilitates pro-inflammatory and prothrombotic conditions (28). sICAM-1 and sVCAM-1 concentrations were elevated in RA (**Table 2**) but were not as strongly associated with disease presence as CRP and SAA.

We also investigated the coagulation profiles of study participants. Türk et al (29). is the only recent study that has assessed thrombotic tendency in RA patient with thromboelastographic assessment (29), using the rotational thromboelastometry (ROTEM). Our findings show that coagulation initiation was amplified in RA patients with shortened velocity parameters of clot formation (R, K, α, TMRTG) (**Figure 2**, **Table 2**). Parameters relating to clot strength (MA, TTG) were attenuated in the RA sample group, but did not statistically differ from healthy controls (**Table 2**, **Figure 2**). Thus, although the blood clots form rapidly it leads to a weak clot.

Excessive hepatic production of fibrinogen is highly prevalent in RA (30), and increased plasma fibrinogen concentration is a strong contributing factor to hypercoagulation. Fibrin(ogen) is susceptible to structural and functional modifications by certain inflammatory molecules, including CRP and SAA (9). Fibrin(ogen) is also prone to post-translational modification that relates to the generation of auto-immunogenicity in RA – the relevance of this process was investigated and is discussed below.

Evaluating fibrin gel matrices visually can reveal much about thrombotic potential under inflammatory conditions. Denser fibrin fiber networks are accompanied with increased resistance to fibrinolysis and is associated with the risk for thrombotic events[reviewed in ref (31)]. Our analysis revealed denser fibrin networks in RA prepared *ex vivo* PPP clots compared to controls (**Figure 3**). This is consistent with a prothrombotic phenotype observed in previous studies that have inspected the fibrin network in RA (10, 11). The average diameter of fibrin fibers was also larger in RA clots compared to controls. Some studies have indicated that thin fibrin fibers have higher tensile strength than thicker fibers, concluding that dense networks consisting of predominantly thin fibers are more resistant to degradation (32). Fibrin networks of this nature in RA were observed by Vrancic et al. (33). However, study by Buclay et al. (34) concluded that thicker fibers are more resistant to plasmin degradation than thinner fibers, owing to their ability to elongate during lysis. It is apparent that our investigation into the structural properties of fibrin networks in RA and its relation to hemostatic function has a rather deceptive appearance.

Distinct protein modifications related to the generation of autoimmunity in RA could present an additional complication when attempting to expound underlying mechanisms responsible for excessive thrombotic risk. Citrullination is a post-translational modification in which positively charged arginine are deiminated by peptidylarginine deiminase (PAD) enzymes to form neutrally charged citrulline (35). Fibrinogen and fibrin are prominent substrates for PAD enzymes and autoantibodies targeting citrullinated fibrin(ogen) have been identified (36–41). The pathogenicity of citrullinated fibrin(ogen) immune complexes have been demonstrated both *in vitro* (13) and *in vivo* (42, 43). Citrullinated fibrin deposits are also common manifestations within synovial cavities, where it contributes to self-perpetuating inflammatory processes (44, 45). Our findings provide novel evidence for the citrullination of fibrin within vasculature which is more prominent in RA plasma compared to control plasma (**Figure 4**). Previously the presence of citrullinated fibrinogen could only be detected in RA synovial fluid (46). Later research by Zhao et al. (39) confirmed the presence of citrullinated fibrinogen containing immune complexes in RA plasma. The insolubility of fibrin may increase the likelihood of it being citrullinated in circulation. The binding of ACPAs to fibrin could then render it less degradable, by decreasing available binding surface to plasmin (47). There remains conjecture as to the effect of citrullination on hemostatic outcome. Citrullination of proteins results in structural unfolding (48) and loss of function (49), which increases its antigenic shelf-life. It has been demonstrated that citrullinated fibrinogen is resistant to thrombin digestion, as preferential epitopes for PADs overlap with thrombin binding sites (15, 16, 50). Despite this, fibrinogenesis in RA is by no means impaired, as evidenced by this study and others. It is plausible that high levels of fibrinogen (30) and thrombin activity (51, 52) in RA has much stronger influence on the fate of fibrinogen than PAD enzymes. There is also evidence that upstream coagulation factors and fibrinolytic components are susceptible to citrullination (53, 54). It is therefore difficult to predict a hemostatic endpoint based on overall citrullination and the effect of citrullination on thrombosis cannot be postulated on singular reactions. The implications that citrullination could have on fibrin, being the end-product of coagulation and a major determinant of thrombotic risk, remains intriguing and should be further investigated.

This study did present some limitations and challenges. The demographic and clinical presentation of the RA patient group was diverse with regards to age and gender distribution, treatment, disease duration and disease severity at the date of sample collection (**Table 1**). The possible confounding effect of antirheumatic drug use on the investigated parameters can be considered less pronounced with a relatively small study sample size as presented here. The strong immunosuppressant effect of both conventional and biologic DMARDs may carry the additional benefit of having cardioprotective properties. However the risk of adverse cardiovascular events have also been related to all currently available RA therapeutics (55). Corticosteroid administration in RA treatment have also been associated with a dose-dependent increase in risk for cardiovascular mortality (56).

## Concluding Remarks

Inflammatory and thrombotic processes are highly pertinent to the development of joint disease and cardiovascular complications. There is a need to study changes that occur within synovial environments in unison with simultaneously occurring changes within circulatory tracts. Further investigation into overlapping processes that are crucially involved in the concurrent development of both RA and CVD could reveal improved global disease markers and novel targets for therapeutic intervention. The formation and structure of fibrin clots in RA shows an atypical pattern compared to conventional observations of hypercoagulation under inflammatory conditions. We propose determining if citrullination causes a structural and functional shift in the nature of fibrin to represent an amyloid-like state. This protein modification could potentially contribute to the formation of aberrant fibrin clots in RA patients that confer a higher degree of thrombotic risk.

## Data Availability Statement

The datasets presented in this study can be found in online repositories. The names of the repository/repositories and accession number(s) can be found at: https://1drv.ms/u/s!Aj29oJ2y_jViqW04QSVHfnjt1YNq?e=qLy8zR.

## Ethics Statement

The studies involving human participants were reviewed and approved by Stellenbosch University HREC committee. The patients/participants provided their written informed consent to participate in this study.

## Author Contributions

JB: Collected, prepared and analyzed the samples and wrote the paper. CV: Technical assistance with fluorescence microscopy. TR: Statistical analysis. GT: Rheumatologist who identified patients, clinical advice. DK: edited the paper. EP: Study leader, funding, edited, and co-wrote the paper. All authors contributed to the article and approved the submitted version.

## Funding

We thank the Medical Research Council of South Africa (MRC) (Self-Initiated Research Program, grant number: A0X331) and the Novo Nordisk Foundation (grant number: NNF10CC1016517) for supporting this collaboration.

## Conflict of Interest

The authors declare that the research was conducted in the absence of any commercial or financial relationships that could be construed as a potential conflict of interest.
